# Characterisation of parapoxviruses isolated from Norwegian semi-domesticated reindeer (*Rangifer tarandus tarandus*)

**DOI:** 10.1186/1743-422X-2-79

**Published:** 2005-09-05

**Authors:** Joern Klein, Morten Tryland

**Affiliations:** 1 Department of Microbiology and Virology, University of Tromsø, Breivika, N-9037 Tromsø, Norway; 2 Danish Institute for Food and Veterinary Research, Department of Virology, Lindholm, DK-4771 Kalvehave, Denmark; 3 Section of Arctic Veterinary Medicine, Department of Food Safety and Infection Biology, The Norwegian School of Veterinary Science, PO Box 6204, N-9292 Tromsø, Norway

## Abstract

**Background:**

Two outbreaks of the disease contagious ecthyma were reported in 1999 and 2000 in Norwegian semi-domesticated reindeer (*Rangifer tarandus tarandus*). Contagious ecthyma is an epidermal disease of sheep and goats worldwide, which is caused by the zoonotic parapoxvirus orf virus. Characterisation of clinical samples from the two outbreaks in semi-domesticated reindeer in Norway by electron microscopy and PCR (B2L) revealed typical parapoxvirus particles and partial gene sequences corresponding to parapoxvirus, respectively. If contagious ecthyma in reindeer is caused by orf virus, the virus may be transferred from sheep and goats, via people, equipment and common use of pastures and corrals, to reindeer. Another possibility is that contagious ecthyma in reindeer is caused by a hitherto unclassified member of the parapoxvirus genus that circulates among reindeer herds and remains endemic in Norway.

**Results:**

Genomic comparisons of one standard orf strain (orf NZ2) and the reindeer isolates, employing restriction fragment length polymorphism (RFLP) and random amplified polymorphic DNA (RAPD) analysis, demonstrated high similarity between the reindeer viruses and known orf virus strains. Partial DNA sequences of two different viral genes were determined for the different isolates and compared with corresponding parapoxvirus genebank sequences. The comparison/alignment and construction of phylogenetic trees also point to an affiliation of the reindeer viruses to the species orf virus.

**Conclusion:**

The results of this work imply that the parapoxvirus causing contagious ecthyma in Norwegian semi-domesticated reindeer belongs to the species orf virus and that the orf virus crosses the host species barrier from sheep and goat to semi-domesticated reindeer.

## Background

Parapoxviruses (PPVs) (family *Poxviridae*) cause dermal diseases most commonly in sheep, goats and cattle [2], but also in semi-domesticated reindeer (*Rangifer tarandus tarandus*) [3] and wildlife, like seals [4], red deer [5] and squirrels [6]. The genus Parapoxvirus consists of five species and three tentative species [7]: Bovine popular stomatitis virus (BPSV), orf virus (ORFV), parapoxvirus of red deer in New Zealand (PVNZ), pseudocowpox virus (PCPV), and squirrel parapoxvirus (SPPV), as well as the tentative species of the genus: auzdyk disease virus, chamois contagious ecthyma virus and sealpox virus.

The first documented outbreak of a contagious ecthyma-like disease in Norwegian reindeer, took place in 1976 among experimental animals at the National Reindeer Research Station in Lødingen [8]. Also human parapoxvirus infections were associated with this outbreak [9]. In April 1999, the first outbreak in reindeer under regular herding conditions was reported in Troms County, followed by an outbreak one year later, also in April, in Nordland County. The outbreak in 2000 involved at least 30 out of 150 reindeer, which were corralled and supplementary fed during the winter. Seven of the infected animals died, due to massive oral cauliflower-like lesions and secondary bacterial infections [1].

In Norway, there are both wild and semi-domesticated reindeer. The latter is herded over an area of approximately 140.000 km^2^, which is about 40% of the mainland area of Norway. Most reindeer herding is conducted by the Saami people (indigenous people of Scandinavia), and the most dense reindeer herding area is in Finnmark county, Northern Norway, where the total number of animals is estimated to be around 140.000 animals (estimate for 2003 [10]). Semi-domesticated reindeer have seasonal migrations between winter (usually inland) and summer (usually coastal areas) pastures, and the pastures are organised in districts, reducing the contact between animals of different districts. However, sheep and goats also use the coastal pastures during spring, summer and autumn, and contact between different animal species is possible. Furthermore, it is not uncommon to use the same corrals and transport vehicles for reindeer and sheep, and remaining scabs from sheep with contagious ecthyma may as well be a source of infection for reindeer. Since the disease contagious ecthyma and orf virus is present among sheep and goats in most parts of the country, it is necessary to characterise the causative agent of contagious ecthyma in reindeer, in order to find out whether reindeer have their own parapoxvirus species, like red deer in New Zealand [5], or whether a transmission of virus between sheep and goats and reindeer is more likely.

Due to genetic heterogeneity within the genus, classification of PPV remains problematic and relies mostly on the source of the virus isolate; id est Parapoxviruses isolated from sheep will be classified as orf virus, virus isolated from cattle as pseudocowpox [11,12]. However, several molecular techniques have been used to characterise parapoxviruses. Inoshima et al. [13] reported a polymerase chain reaction (PCR) protocol assumed to be able to amplify all members of the parapoxvirus genus, based on primers from the B2L gene encoding a major envelope protein [14]. The gene region encoding a viral interleukin 10 orthologue (vIL-10) [15] has been used for characterisation, in combination with sequencing of the amplified DNA products. Restriction fragment length polymorphism (RFLP) has also been used to compare parapoxvirus isolates [5,16], and can be conducted both on genomic DNA as well as on smaller DNA fragments in combination with PCR. Also random amplified polymorphic DNA analysis (RAPD) may be used for characterisation purposes, as reported as a useful technique for comparing orthopoxviruses [17].

The aim of the present study was to characterise the causative agent of contagious ecthyma in semi-domesticated reindeer in Norway, and to compare this virus with other parapoxvirus isolates. This information is necessary to be able to sort out whether reindeer are exposed to orf virus, which is commonly affecting sheep and goats and also present among muskoxen in Norway, to bovine papillar stomatitis virus, which is present among cattle in Norway, or whether reindeer hosts a specific parapoxvirus species.

## Results

### Restriction Fragment Length Polymorphism (RFLP) analysis

**Figure 1:**
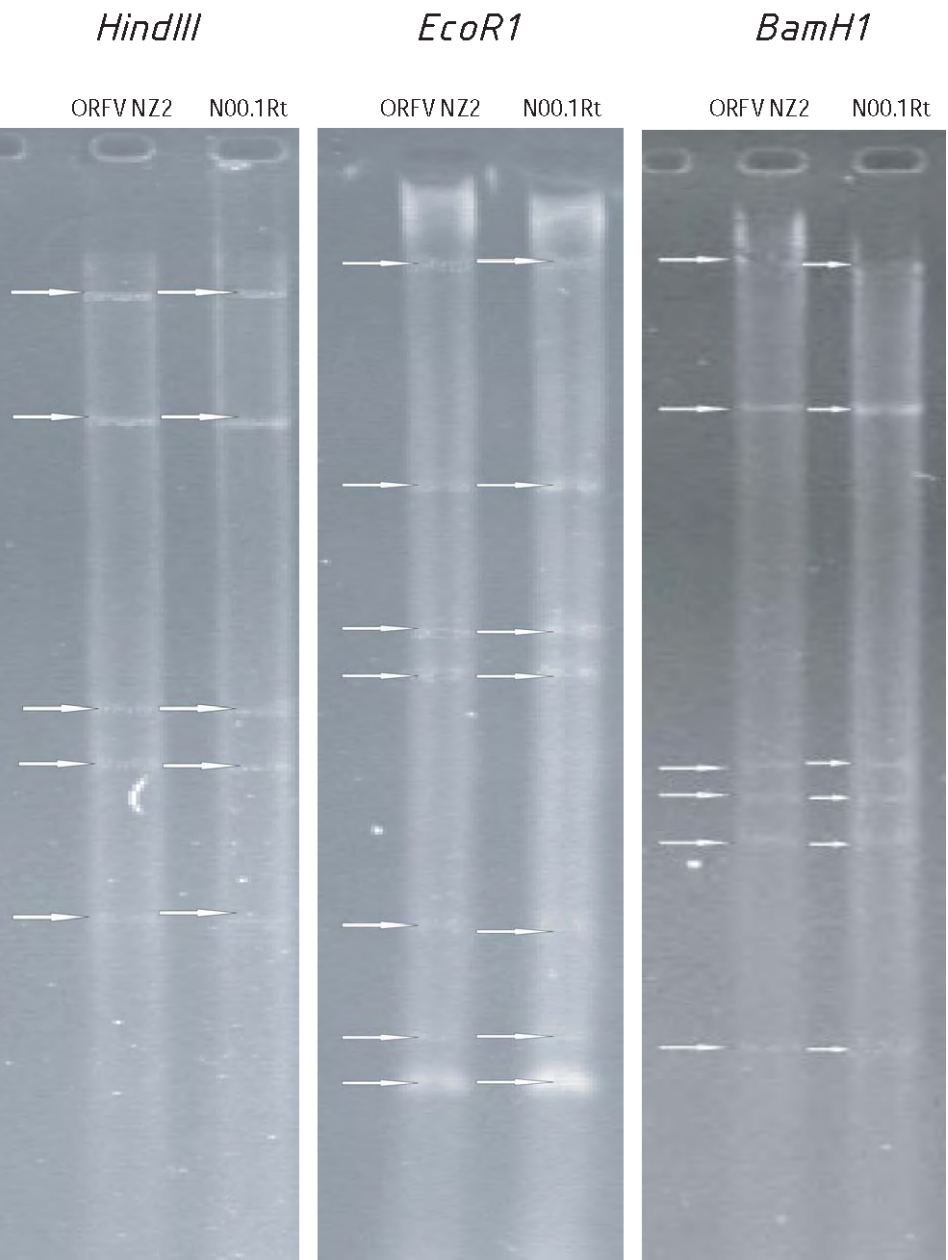
Restriction fragment length polymorphism (RFLP) analysis of the standard orf virus strain NZ2 and the Norwegian reindeer isolate from Troms county 2000 (bands indicated by arrows) displays identical cleavage patterns for the two viruses.

RFLP patterns by the three restriction endonucleases, *Hind III*, *Eco R1 *and *BAM H1 *display identical DNA fragment patterns for orf virus and the parapoxvirus isolated from semi-domesticated Norwegian reindeer (Figure 1).

### Random Amplification of Polymorphic DNA (RAPD) analysis

**Figure 2:**
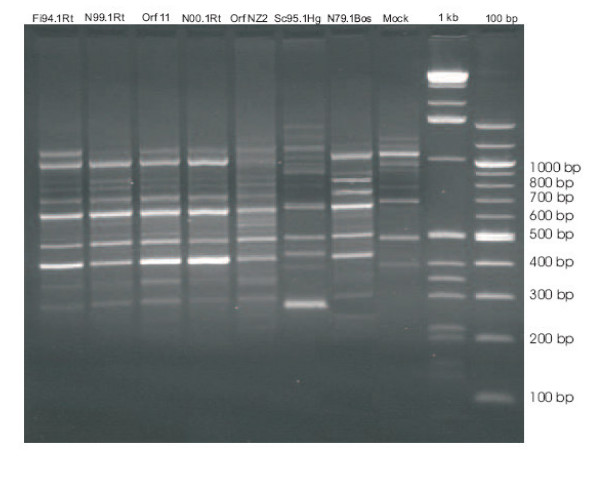
Random amplified polymorphic DNA (RAPD) analysis of different parapoxviruses. Lane 1: reindeer Finland 1994, Lane 2: reindeer Norway 1999, Lane 3: reference strain orf 11, Lane 4: reindeer Norway 2000, Lane 5: orf virus NZ2, Lane 6: parapoxvirus from Weddell seal, Lane 7: parapoxvirus from cattle, Norway, Lane 8: mock infected cells, Lane 9 and 10: 1 kb and 100 bp ladder, respectively. Bands indicate similar patterns for orf and reindeer viruses, whereas parapoxvirus from seal and cattle are different.

The RAPD patterns of the Finnish (Fi94.1Rt) and the Norwegian (N99.1Rt, N00.1Rt) reindeer isolates are similar to that of the orf viruses orf 11 and NZ2 and distinct to the sealpox and pseudocowpox patterns (Figure 2).

### Polymerase Chain Reaction (PCR)

The vIL-10 PCR and GIF PCR were able to amplify DNA from all 25 isolates, whereas the B2L PCR did only amplify DNA from 17 isolates (Table 1).

**Table 1:** PCR and sequencing results obtained from parapoxvirus isolates of different species, reference orf virus strain (Orf 11) and an orf virus vaccine strain (NZ2). Successful amplification is indicated by Genebank accession number

**Signature**	**Host**	**Country of origin**	**B2L-PCR**	**vIL-10-PCR**	**GIF-PCR**
N99.1Rt	*Rangifer t. tarandus*	Norway	AY605963	AY605995	AY605973
N00.1Rt	*Rangifer t. tarandus*	Norway	AY605964	AY605994	AY605972
N00.2Rt	*Rangifer t. tarandus*	Norway	AY605969	AY606005	AY605985
N03.8Rt	*Rangifer t. tarandus*	Norway	AY605966	AY605992	AY606010
Fi94.1Rt	*Rangifer t. tarandus*	Finland	AY605965	AY605993	AY605971
Fi92.1Rt	*Rangifer t. tarandus*	Finland	AY605959	AY606001	AY605979
N79.1Bos	*Bos spec*.	Norway	AY605960	AY606011	AY605980
N92.1Bos	*Bos spec*.	Norway	AY605961	AY606002	AY605981
N83.1Bos	*Bos spec*.	Norway	AY605970	AY606003	AY605982
N85.1Bos	*Bos spec*.	Norway	-	AY963707	AY605984
N71.1Bos	*Bos spec*.	Norway	-	AY606012	AY605983
N02.1Ch	*Capra hircus*	Norway	-	Not sequenced	AY606013
N00.1Ch	*Capra hircus*	Norway	-	AY605999	AY605977
N94.1Om	*Ovibos moschatus*	Norway	AY605962	AY605996	AY605974
N00.1Oa	*Ovis aries*	Norway	AY605957	AY605998	AY605976
N86.1Oa	*Ovis aries*	Norway	AY605968	AY606007	Not sequenced
N86.2Oa	*Ovis aries*	Norway	AY605967	AY606008	AY605990
N03.1Oa	*Ovis aries*	Norway	-	AY963708	AY605991
Sc95.1Hg	*Halichoerus grypus*	Scotland	U49845AJ622901	AY605997	AY605975
N03.1Lw	*Leptonychotes weddelli*	Antarctica	AJ622900	AY606015	AY605989AY605989
Orf 11	*Cell culture*	-	AY605958	AY606000	AY605978
Orf NZ2	*Vaccine*	-	AY963706	AY606006	AY605988

### Phylogenetic analysis

**Figure 3:**
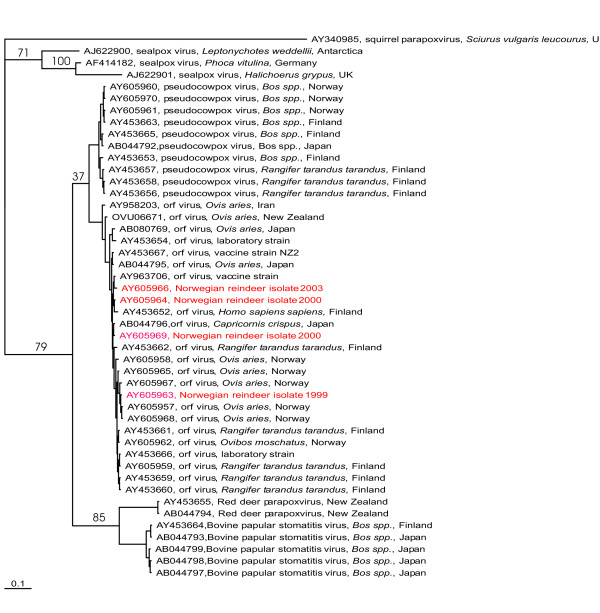
Bayesian tree based on the partial nucleotide sequences of the B2L gene (379 nt) obtained in this study compared with corresponding DNA sequences from parapoxviruses published in Genebank. Isolates are described by Genebank accession number, parapoxvirus species, source and country of origin. Numbers at major clades indicate clade credibility values in Percent.

The Bayesian tree based on the the partial sequences of the B2L-gene (Figure 3) display species specific clustering of the parapoxvirus-genus.

Six clusters, representing the squirrel parapoxviruses, seal poxviruses, bovine papular stomatitis virus, pseudocowpoxviruses, parapoxvirus of red deer in New Zealand, and orf viruses were generated.

The Norwegian reindeer isolates clustered together with orf virus isolates from different host species and geographical origins. Also the Finnish reindeer isolates from 1992 and 1994 were in conjunction with the orf virus isolates, whereas more recent Finnish parapoxvirus isolates causing contagious ecthyma in reindeer have been characterised as more related to pseudocowpoxviruses [18].

**Figure 4:**
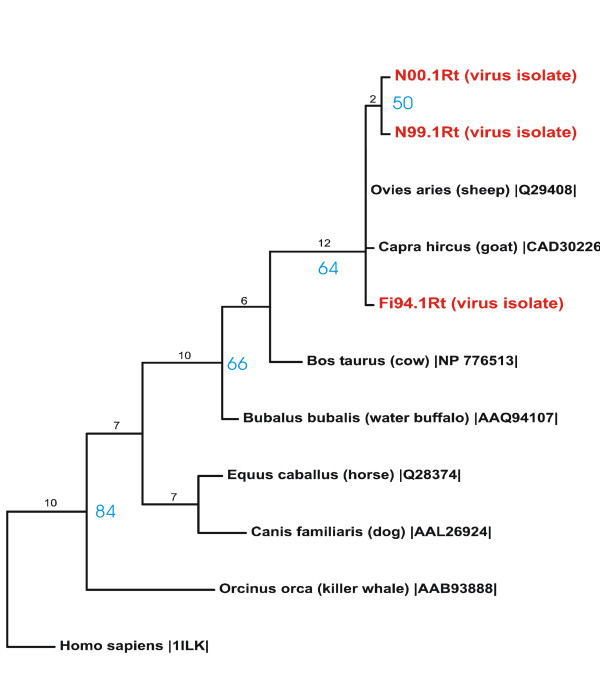
Maximum Parsimony tree based on the translated nucleotide sequences of the Norwegian and Finnish reindeer vIL-10 gene amplicons obtained in this study, compared with corresponding amino acid sequences from mammals published in Genebank. Black numbers (branch length) describe the genetic distance/number of changes along the branch. Blue numbers (bootstrap values) describe the reliability for each clade in percent.

Phylogenetic analysis of interleukin 10 amino acid sequences from a range of mammalian species and the three translated viral interleukin 10 orthologue nucleotide sequences obtained from the two Norwegian (N00.1Rt and N99.1Rt) and one Finnish (Fi94.1Rt) virus isolates from semi-domesticated reindeer is shown in Figure 4. The positions of the viral IL-10 indicate a high similarity to the corresponding genes (interleukin 10) of the main hosts of orf virus, goat and sheep. (Figure 4).

## Discussion

Based on RFLP patterns obtained from the Norwegian reindeer isolates and orf NZ2, a close relationship between these viruses can be assumed (Figure 1). The RAPD patterns of the reindeer isolates and the standard orf virus strains orf 11 and orf NZ2 show high similarity in amplification patterns (Figure 2). This similarity is present in spite of the geographical distance between the Norwegian isolates and the orf strains originating from Great Britain and New Zealand.

Further, this work demonstrates that a virus species characterisation based on the nucleotide sequence of the PCR-product from the B2L-PCR is possible. This characterisation method is easy to perform, because the PCR product is fast to obtain and to proceed.

The phylogenetic analysis of the partial B2L-gene sequence from the Norwegian reindeer isolates shows that these isolates can be allocated to the orf virus species. However, the clade credibility value of 37 % also demonstrate a close relationship between the two different parapoxvirus species, orf and pseudocowpox virus.

Tikkanen et al. [18] demonstrated the affiliation of parapoxvirus isolates obtained from Finnish semi-domesticated reindeer during the early outbreaks (1992–1994) in Finland, to the orf virus species and from later outbreaks to the pseudocowpox viruses, which is congruent with our results.

The close clustering of the translated sequences obtained from virus isolated from reindeer (two Norwegian; 1999, 2000, and one Finnish; 1994) with the amino acid sequences of sheep and goat interleukin 10 demonstrate a high relationship of the parapoxvirus isolated from reindeer to the main hosts of orf virus (sheep and goat). It seems that the viral interleukin 10 orthologue of the reindeer isolates is highly adapted to the immune system of sheep and goats, which also indicates that the reindeer parapoxvirus belongs to the orf virus species.

Our results indicate that neither geographical distance, nor crossing the host barrier from sheep or goat to semi-domesticated reindeer did affect the characteristics of the parapoxvirus orf virus investigated in this study.

The B2L PCR has previously been described as a tool to amplify all species within the parapoxvirus genus [13]. However, we were not able to amplify B2L sequences from nine of the twenty-five isolates included in our study. Many members of the subfamily chordopoxvirinae show genetic rearrangement at the terminal sequences of the genome, which is thought to be an evolutionary mechanism, allowing the virus to adapt to changes of the immune response of the host [19]. Parapoxvirus replicated in vitro show rearrangement of the left terminal end of the viral genome, resulting in the deletion of three genes (E2L, E3L, G1L) and 80 % of a fourth one, the G2L [20-22]. All these four genes play a major role in virulence and host specificity [23]. The B2L gene is localised beside the left terminal end of the genome, so that heterogeneity in the primer binding regions or complete deletion of the B2L gene, through the same rearrangement mechanisms as in the left terminal region itself, can be the reason for the negative results of the B2L-PCR. However, further analyses are needed to evaluate this hypothesis. As compared to the results of the B2L-PCR, the GIF PCR seems to be more sensitive for different members of the genus and can be used for rapid genus-identification. However, the GIF gene is localised in the right terminal gene region [24] and may also undergo genetic rearrangements due to adaptation processes. The GIF gene has so far only been detected in parapoxviruses [23].

The vIL-10-PCR amplified all tested isolates, but rearrangements of the vIL-10 gene have also been demonstrated, in terms of duplications in the inverted terminal repeat [25]. Thus, genetic rearrangements may be a problem when designing a PCR that are supposed to detect parapoxviruses in general. The use of a multiplex PCR, with a combination of one or more of the gene targets desribed above, may thus be a solution to this problem.

## Conclusion

The results of this work point out that the parapoxvirus that has caused contagious ecthyma in Norwegian semi-domesticated reindeer belongs to the orf virus species, and it is to assume that the orf virus crosses the host species barrier from sheep and goat to semi-domesticated reindeer.

Sheep and goats in Norway are commonly free-ranging during the snow-free period, sharing pastures with semi-domesticated reindeer. During seasonal migrations and corralling of animals for slaughter etc. they may also share fences, transport vehicles and other equipment.

As far as we know, contagious ecthyma is the single disease that has caused the most serious economical losses for reindeer herders in the Nordic countries in recent times, and especially so in Finland. Consequently, the common use of equipment, pastures, transport vehicles and facilities for semi-domesticated reindeer, sheep and goats should be avoided, to prevent cross infections.

The outcome of parapoxvirus infections in reindeer seems to be dependent on many environmental factors in addition to the exposure to the virus. Certain types of stress is believed to play a key role, and such stress factors may be lack of food, as well as handling, corralling and transport of animals [3,1]. A changing trend in reindeer herding conditions, facing higher animal densities, faster movements of animals using helicopter and snow mobiles, increased use of supplemental feeding and corralling of animals, and increased transport distances to slaughterhouse may represent factors that can predispose for contagious ecthyma as well as other diseases in reindeer.

## Methods

### Viruses

An overview of the virus isolates included in this study is given in Table 1. Viruses were purified by metrizamide gradient centrifugation from homogenised scab material obtained from Norwegian semi-domesticated reindeer (N99.1Rt, N00.2Rt, N03.8Rt), Finnish semi-domesticated reindeer (Fi94.1Rt, Fi92.1Rt), Norwegian cattle (N79.1Bos, N92.1Bos, N83.1Bos, N71.1Bos, N85.1Bos), Norwegian goat (N02.1Ch, N00.1Ch), Norwegian musk ox (*Ovibos moschatus*) (N94.1Om, N86.1Om), Norwegian sheep (N00.1Oa, N86.1Oa, N86.2Oa, N03.1Oa, N03.2Oa, N00.2Oa), Scottish grey seal (*Halichoerus grypus*) (Sc95.1Hg) and Antarctic Weddell seal (*Leptonychotes weddellii*) (N03.1Lw) as described previously [26].

The orf virus strain orf 11 was provided by the Moredun Research institute (Great Britain) and the orf virus strain NZ2 was derived from a non-attenuated commercial vaccine against contagious ecthyma in sheep (Scabivax^®^, Shering-Plough A/S Animal Health, Norway).

### Cell culture

Orf viruses NZ2 and orf 11 and the parapoxviruses isolated from Norwegian semi-domesticated reindeer (N00.1Rt, N99.1Rt), Finnish semi-domesticated reindeer (Fi94.1Rt) and the Scottish grey seal (Sc95.1Hg) were propagated in Madine-Darby bovine kidney (MDBK; DSMZ No; ACC 174) cells, which are permissive for parapoxviruses [27]. Cells were cultivated in Dulbecco's MEM supplemented with 5 % Fetal Bovine Serum and Penicillin (100 μg/ml)/Streptomycin-solution (100 IU/ml) and incubated at 37°C with 5 % CO_2_.

### DNA extraction

For the purpose of RFLP and RAPD analysis, viral DNA was extracted from the cytoplasma of infected cells, using reducing, non-ionic and proteolytic detergents, as described in detail by Esposito et al. [28].

For PCR, viral DNA was extracted using QIAamp^® ^DNA Mini Kit (QIAGEN, Hilden, Germany).

### RFLP

Viral DNA was digested for 4 hours with the restriction enzymes *Hin*dIII, *Eco*RI and *Bam*HI (NEW ENGLAND BioLabs^®^Inc., UK). DNA fragments were separated on a 0.6 % agarose gels for 20 hours at 0,6 V/cm.

### RAPD

RAPD were conducted with the commercial kit Ready.To.Go^® ^RAPD Analysis Beads (Amersham Biosciences AB, Uppsala, Sweden). Five μl of the RAPD analysis primer no. 6 (5'- CCCGTCAGCA-3') were added to 19 μl of dH2O and 1 μl of template DNA and gently mixed. The following low stringency cycling profile was used: initial denaturation at 95°C for 4 min, followed by 45 cycles consisting of denaturation at 95°C for 1 min, annealing at 36°C for 1 min, and elongation at 72°C for 2 min. DNA fragments were separated on a 2 % agarose gel at 7,5 V/cm for 2,5 hours.

### PCR

Three different PCR protocols were performed as specified below:

#### B2L-PCR

Inoshima et.al. [13] describe a PCR specific for the detection of all parapoxviruses, resulting in the amplification of theoretically a 594 bp product. The primers (PPP 1 and PPP 4) are based on the B2L gene sequence of the orf virus strain NZ2. The B2L gene encode a homologue of the vaccinia virus major envelope antigen p37K gene [29]. PCR was carried out as described by Inoshima et al. [16] with the exception that 5 % dimethylsulfoxide (DMSO) was added to the reaction mix as a PCR enhancer.

#### GIF-PCR

The Granulocyte-macrophage-colony-stimulating factor (GM-CSF) and Interleukin-2 inhibition factor (GIF) is found only in parapoxviruses, and represents an important virulence factor [24]. Amplification of parts of the GIF gene may thus be useful, both for detection of parapoxvirus DNA in tissue samples and for virus species differentiation. The GIF PCR primers (GIF 5 → 5'-gct cta gga aag atg gcg tg-3' GIF 6 → 5'-gta ctc ctg gct gaa gag cg -3'), generating amplicons of approximately 408 bp, were obtained from the published sequence of the orf virus GIF gene (Genebank accession number AF192803.1; Deane et al., 2000) and selected by the online tool "GeneFisher" [30].

Five μl template DNA were added to 45 μl of the PCR reaction mixture containing 0.2 mM primers (GIF 5 and GIF 6), 200 mM each of dATP, dCTP, dGTP and dTTP, 10 mM Tris-HCl (pH 8.3), 50 mM KCl, 1.5 mM MgCl_2 _and 1 U of AmpliTaq^® ^Gold DNA polymerase (Applied Biosystems, Norway). DNA was amplified with a DNA Thermal Cycler PE9700 (Perkin Elmer) by a two-step cycling reaction as follows: 95°C for 15 min, and five cycles of 94°C for 30 sec, 57°C for 2 min and 72°C for 30 sec, and then 35 cycles of 94°C for 30 sec, 57°C for 30 sec and 72°C for 30 sec, followed by a final extension step of 72°C for 10 min. The resulting PCR product was examined by electrophoresis, using a 1,2 % agarose gel, containing 0,005 % ethidium bromide, with a separation time of 1,5 hours at 6,5 V/cm.

#### vIL-10 PCR

The viral interleukin 10 orthologue [15] need to have a close similarity to the IL-10 of the host for effective virus propagation. PCR targeting this gene may be useful for genus affiliation, and nucleotide sequencing of the PCR amplicon followed by virtual translation to the protein sequence may be suitable for virus characterisation.

Primers (vIL-10-3 → 5'-atg cta ctc aca cag tcg ctc c-3', vIL-10-4 → 5'-tat gtc gaa ctc gct cat ggc c-3') were obtained from consensus sequences previously reported to Genebank (accession numbers OVU82239; [31], OVU60552; [32], AY231116.1; [33], AY186733.1; [34], AY186732; [34]) and selected by the online tool "GeneFisher[30]. The expected length of the resulting amplicon is approximately 300 bp. The reaction mixture, cycling profile and agarose gel analysis was conducted as described for the GIF PCR.

### Nucleotide sequencing

The resulting amplicons of the B2L, GIF and vIL-10 PCR were prepared for nucleotide sequencing by enzymatic removal of unused dNTP and primers (ExoSAP-IT™; Amersham Pharmacia Biotech, Sweden). The enzyme preparation (0,5 μl; ExoSAP-IT™) was added directly to 6,5 μl of the PCR product and incubated at 37°C for 1 hour. ExoSAP-IT™ was inactivated by heating to 80°C for 15 minutes. After the clean-up procedure the sequencing protocol for the BigDye^®^Terminator v3.1 cycle sequencing kit (Applied Biosystems, Norway) was performed. Seven μl of the purified PCR product was mixed with 4 μl "Ready Reaction Premix", 2 μl sequencing-buffer, 3,2μl of 20 μM Primer solution, 1 μl DMSO and 2,8 μl dH2O. This mixture was thermal cycled 25 times at 96°C for 10 seconds, 50°C for 5 seconds and 60°C for 4 minutes. DNA was precipitated with ethanol and the sequence was determined with the "ABI PRISM 377 Genetic Analyser" (Applied Biosystems, Norway).

### Phylogenetic analysis

Multiple sequence alignment of the 379 nucleotide long partial B2L-sequence was conducted using CLUSTAL X (version 1.81; [35]) and phylogenetic analysis was performed by Bayesian Analysis using MrBayes [36] with the following settings. The maximum likelihood model employed 2 substitution types ("nst = 2"), with base frequencies set to the empirically observed values ("basefreq = empirical"). Rate variation across sites was modelled using a gamma distribution (rates="gamma"). The Markov chain Monte Carlo search was run with 4 chains for 500000 generations, with trees begin sampled every 100 generations (the first 1000 trees were discarded as "burnin").

## Competing interests

The author(s) declare that they have no competing interests.

## Authors' contributions

This work is based on the MPhil thesis of Jörn Klein. Morten Tryland was the main supervisor of this thesis and project leader. The thesis is available online under: 
